# Optimizing Clinical Cardiac MRI Workflow through Single Breath-Hold Compressed Sensing Cine: An Evaluation of Feasibility and Efficiency

**DOI:** 10.3390/jcm13030753

**Published:** 2024-01-28

**Authors:** Fuyan Wang, Junjie Zhou, Cailing Pu, Feidan Yu, Yan Wu, Lingjie Zhang, Siying Ma, Hongjie Hu

**Affiliations:** 1 Department of Radiology, Sir Run Run Shaw Hospital, Zhejiang University School of Medicine, 3# East Qingchun Road, Hangzhou 310016, China; wfylink@zju.edu.cn (F.W.); junjiezhou@zju.edu.cn (J.Z.); cailingpu@zju.edu.cn (C.P.); y214180937@zju.edu.cn (F.Y.); wuyan0526@zju.edu.cn (Y.W.); y216180175@zju.edu.cn (L.Z.); 22218723@zju.edu.cn (S.M.); 2 Department of Radiology, The Fourth Affiliated Hospital, International Institutes of Medicine, Zhejiang University School of Medicine, 1# Shangcheng Avenuee, Yiwu 322000, China

**Keywords:** cardiac magnetic resonance, compressed sensing, flip angle, examination workflow

## Abstract

Background: Although compressed sensing (CS) accelerated cine holds immense potential to replace conventional cardiovascular magnetic resonance (CMR) cine, how to use CS-based cine appropriately during clinical CMR examinations still needs exploring. Methods: A total of 104 patients (46.5 ± 17.1 years) participated in this prospective study. For each participant, a balanced steady state free precession (bSSFP) cine was acquired as a reference, followed by two CS accelerated cine sequences with identical parameters before and after contrast injection. Lastly, a CS accelerated cine sequence with an increased flip angle was obtained. We subsequently compared scanning time, image quality, and biventricular function parameters between these sequences. Results: All CS cine sequences demonstrated significantly shorter acquisition times compared to bSSFP_ref_ cine (*p* < 0.001). The bSSFP_ref_ cine showed higher left ventricular ejection fraction (LVEF) than all CS cine sequences (all *p* < 0.001), but no significant differences in LVEF were observed among the three CS cine sequences. Additionally, CS cine sequences displayed superior global image quality (*p* < 0.05) and fewer artifacts than bSSFP_ref_ cine (*p* < 0.005). Unenhanced CS cine and enhanced CS cine with increased flip angle showed higher global image quality than other cine sequences (*p* < 0.005). Conclusion: Single breath-hold CS cine delivers precise biventricular function parameters and offers a range of benefits including shorter scan time, better global image quality, and diminished motion artifacts. This innovative approach holds great promise in replacing conventional bSSFP cine and optimizing the CMR examination workflow.

## 1. Introduction

Cardiac magnetic resonance (CMR) is a dependable and precise method for quantifying cardiac function, particularly for ventricular volume and ejection fraction. These parameters are commonly utilized to formulate treatment strategies for patients with various heart diseases [1,2]. The retrospective electrocardiogram (ECG)-gated breath-hold balanced steady state free precession sequence (bSSFP) cine is a crucial CMR sequence for obtaining quantitative parameters and morphological changes. However, its long breath-hold time and propensity for artifacts can lead to increased scanning time [3]. This prolonged acquisition time can increase the likelihood of body motion, decrease global image quality and completion ratio of clinical CMR examinations [4]. To overcome this constraint, it is essential to employ a promising acceleration method to reduce the duration of the long-axis and multi-slice whole ventricular coverage short-axis cine scan [5].

Compressed sensing (CS) is a rapidly advancing magnetic resonance technology, which innates an essential way to reduce acquisition time by applying highly under-sampled k-space and iterative reconstruction in recent years [6,7]. CS technique leverages the sparsity of images in a transform domain, enabling the reduction of aliasing artifacts resulting from random undersampling through a nonlinear iterative reconstruction process [8]. When combined with parallel imaging, the acceleration rates of the CS-based cine sequence can reach a comparable range to real-time cine [9]. Although the application of CS imaging in MRI has been in use for over a decade, properly integrating it in complex clinical CMR examinations can still pose challenges. Utilizing a reliable CS cine sequence and appropriate examination workflow may outweigh the use of conventional bSSFP cine alone for patients with diverse heart disease etiologies [10].

The primary objective of this study is to optimize the clinical CMR examination workflow through the use of CS-based cine which allows for the acquisition of 8–12 short-axis slices during a single breath-hold (abbreviated to CS-cine in the following context). Assessing the reliability of CS-cine compared to the routine employed bSSFP cine sequences.

## 2. Materials and Methods

### 2.1. Study Population

**Figure 1:**
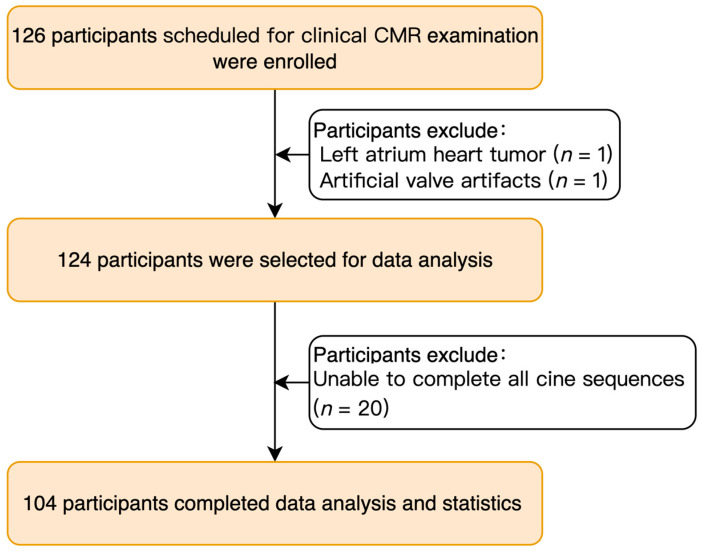
The patients’ enrollment flowchart. CMR, cardiovascular magnetic resonance.

From January 2022 to August 2022, we prospectively enrolled 126 patients routinely scheduled for clinical CMR in this study. Prior to the CMR examination, all patients underwent a pre-evaluation to ensure their renal function (eGFR: >40 mL/min), heart rate (less than 90 bpm), and breath-hold ability (more than 10s duration) were suitable for the examination. Cine image quality was manually checked after scanning. Two patients were excluded from the data analysis, respectively due to a left atrium tumor and artificial valve related artifacts. Additionally, 20 patients who were unable to complete all cine sequence scans due to poor breath-holding capacity or unpredictable arrhythmias were also excluded. Of these 20 individuals, 16 patients were excluded partly because the scan was stopped because the multislice bSSFP sequences could not be acquired due to the inability to hold their breath during the scan or because of heart rate irregularities (most of the patients showed a change in heart rhythm caused by breath-holding), the others were excluded because they continued to be scanned due to the presence of CS-cine, but at last, four sets of cine sequences were incomplete and the subsequent analysis was unenforceable. Four patients who were overweight (BMI greater than 30 kg/m^2^) were excluded because they had large banding artifacts in the CS-cine images that prevented the assessment of cardiac function. (The flow chart of the study is shown in Figure 1). Ultimately, 104 patients were included in the final analysis. This study was conducted in accordance with the Declaration of Helsinki and received approval from the Ethics Committee of our Hospital (approval number: 2022–0212). Written consent was obtained from all participants or their surrogates (two patients were under the age of 18).

### 2.2. MR Image Acquisition

**Figure 2:**
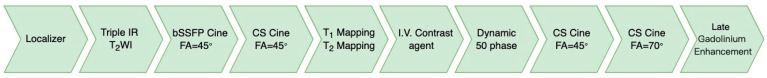
CMR scanning procedure. IR, Inversion Recovery; CS, compressed sensing; bSSFP, balanced steady state free precession.

All CMR examinations were performed on a 1.5 T MRI scanner (uMR 680, United Imaging Healthcare, Shanghai, China), equipped with a 24-channel dedicated cardiac coil and high-speed reconstruction hardware. Four standard views of the heart (two chamber, four chamber, three chamber, and short-axis) were captured for all cine sequences. The short-axis cine consisted of 8–12 slices, aligned with the mitral valve and covering the entire left ventricle. Each cine slice comprised 25 phases. The routine employed bSSFP cine uses GRAPPA (Generalized Autocalibrating Partially Parallel Acquisitions) in parallel acquisition techniques with an acceleration factor 2. Each patient underwent four series of standard view cine scanning: first, the referenced retrospective ECG-gated conventional multi-breath-hold segmented bSSFP cine sequence (bSSFP_ref_) was scanned; then, the CS-cine sequence with 45° flip angle was acquired (CS_45_); after enhancement agent injection (Gadodiamide, 3.0 mL/s, 0.15 mmol/kg), the CS-cine sequence with the same parameters as CS_45_ (eCS_45_) and the CS-cine with an increased flip angle (70°, eCS_70_) was acquired. The detailed scanning procedure and imaging parameters are summarized in Figure 2 and Table 1.

### 2.3. Ventricular Volume Assessment

All eligible cine CMR images were analyzed on commercially available CVI^42^ software (Circle Cardiovascular Imaging, Calgary, Alberta, Canada) by one experienced radiologist who was blinded to the type of cine images (FYW, 7 years of CMR experience). LV and RV volumes, as well as myocardial mass, were evaluated after drawing the contours of endocardial and epicardial borders during the end-diastole and end-systole phases on the cine short-axis stack semi-automatically (manual correction of the contours was needed occasionally), in adherence with the Society for Cardiovascular Magnetic Resonance’s post-processing guidelines [11]. The stroke volume (SV) was computed as the disparity between the end-diastolic volume (EDV) and end-systolic volume (ESV), whereas, the ejection fraction (EF) was calculated as SV divided by EDV multiplied by 100. The reproducibility of left ventricular volume assessment was investigated by two independent blinded observers in 20 randomly selected subjects (YW and CLP, 3 and 5 years of CMR experience separately). For the assessment of intra and inter-observer reproducibility, an interval of six weeks was chosen between the first and second analyses.

### 2.4. Qualitative Image Scoring

Two experienced CMR specialists (FYW and CLP) jointly assessed the image quality. If a consensus was not reached, a third specialist (YW) was consulted to resolve any discrepancies. Anonymous short-axis cine images from four different cine sequences were presented, and global image quality and artifact scoring were stratified across three and five categories, respectively. For diagnostic purposes, global image quality was divided into three levels, including poor (non-diagnostic)—1, adequate (diagnostic)—2, and good (diagnostic)—3 [12]; Five levels were established to score artifacts, adapted from the EuroCMR standardized criteria [13]. A rating of 5 meant the absence of artifacts. A score of 4 indicated artifacts (wrap-around, respiratory or cardiac ghosting, blurring, metallic susceptibility, or shimming) that affected the clarity of more than one-third of the left ventricle endocardial border during end-diastole or end-systole on a single short-axis slice. A rating of 3 denoted artifacts affecting two slices (following the aforementioned rules as score 4), while the same law applied to 2 (affecting three slices) and 1 (more than three slices) point [14]. Artifact scores less than 3 were considered not available for biventricular function analysis. All the cine sequences of these patients eventually enrolled in this research exhibited a global image quality score exceeding 1, to fulfill the requirements of clinical diagnosis.

### 2.5. Image Contrast Evaluation

Image contrast was quantitatively described as a blood pool–to–myocardial signal intensity ratio. The blood pool and myocardial signal intensity values were measured in the end-diastolic midventricular short-axis cine image in all patients. The myocardial signal intensity was the mean pixel intensity in a circular region of interest placed in the middle septum. The region of interest had a diameter about two-thirds the width of the septum. The blood pool signal was measured by using a same-sized region of interest through four cine series placed in the center of the LV cavity. The formula was: SI_pool-to-myo_ = SI_pool_/ SI_myo_ (SI, signal intensity) [14].

### 2.6. Statistical Analysis

Statistical analysis was conducted using IBM SPSS (v. 26.0, Armonk, NY, USA) and GraphPad Prism (v. 9.0, Boston, MA, USA). Mean ± standard deviation (SD) was used to express all normally distributed continuous data, while categorical variables were presented as counts or percentages. After confirming normal distribution using the Shapiro-Wilk test, paired t-test and Bland-Altman analyses (the data were collected from the same individual) were employed to compare the differences in scan time and cardiac function parameters between each pair of cine sequences. The Wilcoxon matched-pairs signed-rank test was used to compare image quality between every two cine sequences. Meanwhile, intraclass correlation efficiency (ICC) was utilized to evaluate inter and intra-observer consistency in assessing the reproducibility of left ventricular functional parameters. A *p* value less than 0.05 (two-tailed) was considered statistically significant.

## 3. Results

### 3.1. Demographic

A total of 104 recruited patients (75 male, 29 female; mean age: 46.5 ± 17.1 (SD) years; age range: 14–86 years) completed all cine sequences. The etiologies they applied for CMR examination included hypertrophic cardiomyopathy (*n* = 24), arrhythmia (*n* = 23), ischemic cardiomyopathy (*n* = 17), dilated cardiomyopathy (*n* = 13), myocarditis (*n* = 11), hypertension (*n* = 5), amyloidosis (*n* = 3), takotsubo cardiomyopathy (*n* = 2), rheumatic heart disease (*n* = 3), arrhythmogenic right ventricular cardiomyopathy (*n* = 1), glycogen shortage disease (*n* = 1) and sarcoidosis (*n* = 1) (details shown in Table 2).

### 3.2. Scanning Time and Biventricular Function

**Figure 3:**
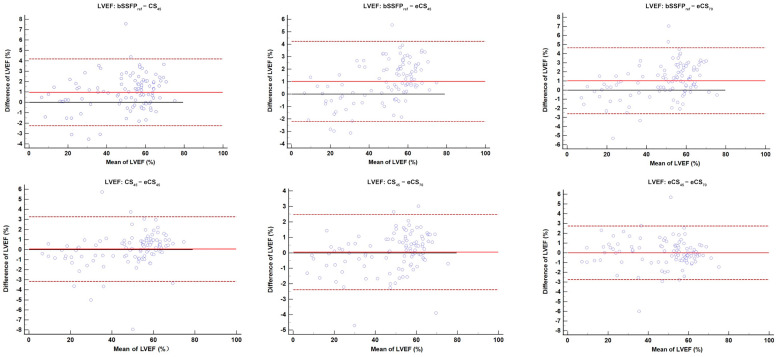
A Bland-Altman plot analysis revealed discrepancies in the LVEF. bSSFP_ref_ cine exhibited a slightly elevated LVEF when compared to all CS-cine sequences (*p* < 0.001). However, there was no significant variation in LVEF detected among the three CS-cine sequences. LVEF, left ventricular ejection fraction; CS, compressed sensing; bSSFP, balanced steady state free precession. CS_45/70_ = CS-cine with 45/70° flip angle, e represents contrast enhanced.

Compared to bSSFP_ref_ cine, short-axis CS-cine sequences need significantly shorter acquisition time (18.2 ± 3.2 (SD) s vs. 119.7 ± 23.3 (SD) s, *p* < 0.001), not considering the additional time consumption for breath-holds and voice commands ((7–11 times depending on the size of the left ventricle, 10s each time, bSSFP_ref_ cine scan duration reach 210.6 ± 25.5 s totally). Shorter acquisition times were also observed in the long-axis CS-cine sequences (1.8 ± 0.3 (SD) s vs. 10.5 ± 2.4 (SD) s, *p* < 0.001). The heart rates for all participants in four cine sequences (short-axis) showed no significant difference (67.7 ± 11.8 (SD) bpm, detailed in Table 3). bSSFP_ref_ cine showed a slightly higher left ventricular ejection fraction (LVEF) compared to all CS-cine sequences (bSSFP_ref_: 49.2% ± 16.9% (SD); CS_45_: 48.3% ± 16.5% (SD); eCS_45_: 48.2% ± 16.1% (SD); eCS_70_: 48.2% ± 16.1% (SD), all *p* < 0.001). The mean differences were close to 1.0% (bSSFP_ref_ vs. CS_45_, 95% CI: 0.6% to 1.3%; bSSFP_ref_ vs. eCS_45_, 95% CI: 0.7% to 1.3%; bSSFP_ref_ vs. eCS_70_, 95% CI: 0.7% to 1.4%). There were no statistical differences in LVEF among the three CS-cine sequences (see Figure 3). The right ventricular ejection fraction (RVEF) for two enhanced CS-cine sequences was lower than the two unenhanced cine sequences (bSSFP_ref_: 42.9% ± 13.5% (SD); CS_45_: 42.8% ± 13.5% (SD); eCS_45_: 41.8% ± 13.2% (SD); eCS_70_: 42.0% ± 13.0% (SD), all *p* < 0.001). For right ventricular stroke volume (RVSV), CS_45_ was significantly lower than bSSFP_ref_ (bSSFP_ref_: 57.8 ± 26.1 mL (SD); CS_45_: 56.9 ± 25.8 mL (SD), *p* < 0.05) while both RVSV for two enhanced CS-cine sequences was higher than the two unenhanced cine sequences (bSSFP_ref_: 57.8 ± 26.1 mL (SD); CS_45_: 56.9 ± 25.8 mL (SD); eCS_45_: 60.4 ± 26.7 mL (SD); eCS_70_: 60.2 ± 26.0 mL (SD), all *p* < 0.001). End-systolic/diastolic volume (ESV/EDV) for both ventricles increased after contrast agent injection (all *p* < 0.001), but no significant difference was observed between eCS_45_ and eCS_70_ cine (detailed in Table 4).

### 3.3. Image Quality

**Figure 4:**
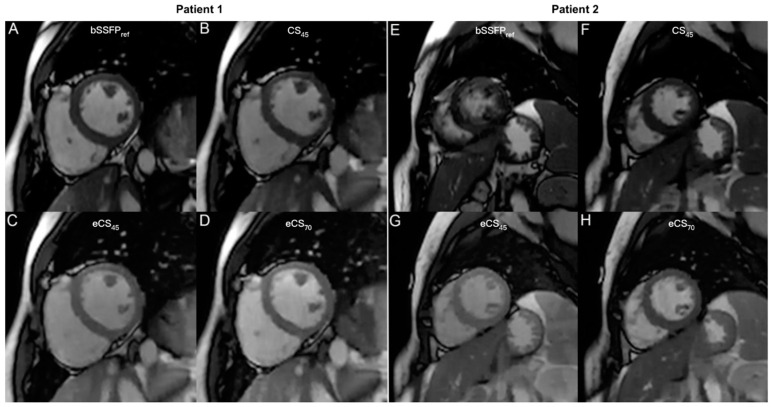
Images of conventional bSSFP_ref_ cine and CS-cine in two patients. Patient 1 was a 33-year-old man with paroxysmal arrhythmia. The diastole phase of the conventional bSSFP_ref_ and CS-cine sequences are presented in panels (**A**–**D**). All cine images exhibit excellent image quality (global image quality = 3, artifact score = 5). Patient 2 was a 52-year-old woman with ischemic cardiomyopathy, and the same sequences as patient 1 are shown in panels (**E**–**H**). Notably, significant artifacts were observed in the bSSFP_ref_ cine images due to a short breath-hold duration (global image quality = 2, artifact score = 3), while all CS cine images provided better quality images. CS, compressed sensing; bSSFP, balanced steady state free precession. CS_45/70_ = CS-cine with 45/70° flip angle, e represents contrast enhanced.

**Figure 5:**
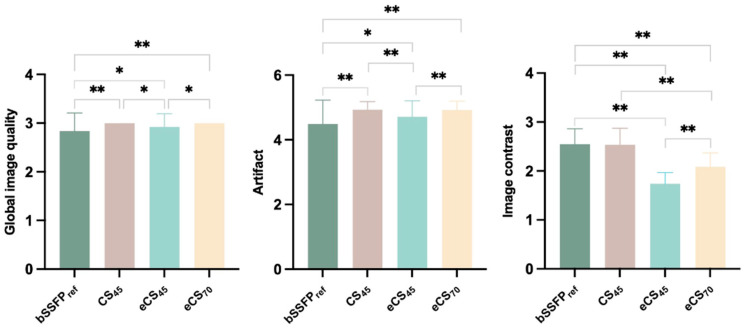
Comparison of global image quality, artifact score, and image quality among conventional bSSFP_ref_ and CS-cine sequences. * = *p* < 0.05, ** = *p* < 0.001. CS, compressed sensing; bSSFP, balanced steady state free precession. CS_45/70_ = CS-cine with 45/70° flip angle, e represents contrast enhanced.

Independent of scanning parameters, all CS-cine exhibited higher global image quality (*p* < 0.05) and fewer artifacts than bSSFP_ref_ cine (*p* < 0.005), unenhanced CS_45_ and enhanced eCS_70_ cine sequences showed no difference in global image quality and artifact score. CS_45_ and eCS_70_ cine displayed higher global image quality than eCS_45_ cine (*p* < 0.005). Conventional bSSFP_ref_ and unenhanced CS cine sequences presented the same image contrast, meanwhile, the gadolinium agent decreased the image contrast of CS-cine sequences regardless of the flip angle rising (*p* < 0.001) (see Figure 4 and Figure 5).

The inter and intra-group consistency of left ventricular function that derived from bSSFP_ref_ and three CS-cine sequences were all higher than 0.90 (all *p* < 0.001, details shown in Table 5, Supplementary Tables S1 and S2).

## 4. Discussion

In this study, CS-cine demonstrated its potential in refining workflow by replacing conventional cine in patients with various heart diseases. We found CS-cine could dramatically reduce scanning duration, provide reliable volume function parameters, maintain similar image contrast compared with referenced conventional cine, and provide better diagnostic image quality in our patient cohort. Increased flip angle could some degree compensate for the influence of gadolinium contrast agent, however, CS-cine is still recommended to be used before contrast injection.

How to accelerate the speed and reduce the artifact of CMR examination become a major research direction in recent years. Several previous studies focused on testing the feasibility and stability of CS-based sequences in certain kinds of heart disease [9,15]. However, the clinical applicability of CS cine largely depends on establishing a flexible and efficient workflow for patients with different cardiac vascular diseases, taking into account not only the patient’s breath-hold ability and physical state, but also its ability to provide reliable cardiac function parameters and image quality [1].

By combining the CS acceleration technique with bSSFP cine in four standard cardiac views (2/3/4 chamber and short-axis), a significant reduction of scanning time was observed in CS-cine sequences, particularly in the multi-slice short-axis view. This highlights the effectiveness of the sparse sampling and iterative reconstruction CS technique in expediting CMR imaging [16]. For patients in a weak state and requiring shorter CMR examinations, or having short breath-hold time to complete routine bSSFP short-axis cine, CS cine could be a more viable option to accelerate the CMR examination procedure [17].

Moreover, when contemplating the replacement of traditional balanced steady state free precession (bSSFP) cine, it is essential to consider factors beyond the speed of image acquisition. Equally significant is the assessment of whether CS-cine can provide comparable assessments of heart function as bSSFP cine. LVEF holds a central position in measuring left ventricular systolic function within all parameters of LV function assessment. Its routine utilization in guiding clinical decisions, device therapies, and interventions, including surgeries, highlights its importance [18]. Although our result showed that the LVEF obtained from bSSFP_ref_ cine was significantly higher than CS-cine sequences, the mean differences were all extremely close to 1.0%, which manifested an almost negligible impact on clinical judgment of cardiac function [17,19,20]. In the bSSFP_ref_ group, there were 22 patients with LVEF < 35%, while in the CS_45_ group, there were 24, indicating that CS-cine sequences exhibit satisfactory diagnostic performance, preventing the oversight of such patients. Accordingly, CS-cine sequences could be used in patients with diminished LVEF, which may not affect the patient’s clinical management. We noted significant changes in LVEDV and LVESV between each of the cine sequences, with unenhanced CS-cine underestimating LVEDV compared to the conventional method, as seen in previous studies, this discrepancy might be attributable to temporal regularization [21]. Remarkably, there were no notable variations in LVEF detected amongst all CS-cine sequences. This discovery strengthens our conviction that LVEF is a more consistent heart function indicator than LVEDV and LVESV, even when taking into account the presence of dilation or ventricular impairment in some study participants. The flip angle and gadolinium contrast agent are unlikely to impact it in CS-cine sequences (see Figure 3). RVEF exhibited equivalent dependability as LVEF, despite notable variations in RVEDV and RVESV. The injection of gadolinium agent reduced the RVEF value of CS-cine sequences, but the compromised spatial resolution and augmented flip angle had no bearing on its accuracy. Although LV mass followed a similar path as RVEF, significant differences were observed between all cine sequences except for bSSFP_ref_ and eCS_70_. The comparison results indicate that CS-cine could reliably provide biventricular function indexes, such as LVEF and RVEF, similar to routine bSSFP cine. Additionally, it was discovered that the potential of CS-cine was unaltered by adjustments in flip angle or the administration of gadolinium contrast.

**Figure 6:**
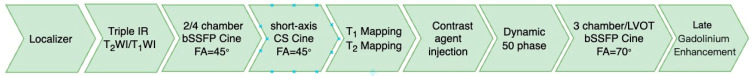
Refined CMR scanning workflow of our 1.5 T MR scanner. CS, compressed sensing; bSSFP, balanced steady state free precession. FA, flip angle. LVOT, Left ventricular outflow tract. IR, inversion recovery.

Image quality also plays a significant role, directly impacting the routine application possibility of CS cine [22]. Based on our observations, undiagnostic bSSFP_ref_ cine images resulted in the exclusion of 15 patients (while all CS-cine provided diagnostic images), whereas only four patients were excluded due to undiagnostic CS-cine images. Notably, all undiagnostic CS-cine images originated from patients with a body mass index (BMI) greater than 30 kg/m^2^. Our study uncovered that CS-cine images exhibited fewer artifacts and exhibited global image quality that was at least equal to, if not superior to, bSSFP_ref_ in patients with various etiologies. This may contrast with previous findings that conventional bSSFP sequences offered equal or better image quality than CS-based cine sequences [23,24]. Several explanations may be offered: first, the patient cohort in our study, which included 37 patients diagnosed with heart failure (21 of whom were classified as NYHA (New York Heart Association) II, 14 as NYHA III-IV); and second, the inherent robustness of CS imaging, which can tolerate poor breath-holding and is less dependent on flip angle and contrast agent administration [25]. While the CS-cine sequence displayed a similar image contrast to the conventional bSSFP_ref_ cine sequence, it demonstrated a drawback in displaying moving valves in long-axis views, which somewhat limited the applicability of CS-cine in those perspectives. Moreover, flip angles and gadolinium agents could potentially impact image contrast, a finding consistent with previous research [26]. Following the administration of a contrast agent, the image contrast in CS-cine was observed to diminish. Nevertheless, an augmentation in the flip angle mitigated this decline to some extent. This suggests that employing a larger flip angle can contribute to generating cine images of similar quality to conventional bSSFP cine sequences following contrast agent injection. However, it is crucial to account for the specific absorption rate (SAR) when deciding on the degree of flip angle. Our choice of flip angle degree was determined via pretests (ranging from 55 to 105°) on several patients, taking into consideration both image contrast and SAR. Based on the obtained results and previously published findings, we have refined the CMR examination procedure for our 1.5 T MR scanner, which is routinely used for cardiac examinations, especially for patients suffering from heart failure or in a critical state. Based on the aforementioned findings, a revised procedure has been outlined in detail in Figure 6. In our future clinical CMR examinations, we will continue to assess the feasibility of this procedure, aiming to facilitate the implementation of CS-cine in all of our MR scanners equipped with CS technology.

The study was subject to several limitations. Firstly, the data was solely collected from a single clinical center, which may have introduced bias in the demographics and clinical practices of the participants. Secondly, accurately controlling the effect of the gadolinium agent on CS-cine images was difficult due to the varying agent injection volumes of different patients. Thirdly, during the scanning process, the effective temporal resolution of this single breath-hold CS-cine sequence was 42.9 ms, which was higher than that of bSSFP_ref_ (31.2 ms). Hence, the obtained cardiac phases may diverge somewhat from the standard bSSFP cine sequence, potentially impacting the assessment of biventricular volume and function. In the future, research endeavors should encompass a broader spectrum of patients to corroborate our pivotal discoveries and advocate for the widespread adoption of the CS technique in all clinical CMR examinations.

## 5. Conclusions

The CS-cine sequence provides a reliable method for obtaining cardiac function and image quality parameters in patients with various causes of heart disease, while significantly reducing scanning time. It possesses great potential for replacing routine bSSFP cine and refining CMR examination workflow. It is strongly recommended to utilize this CS-cine sequence in a short-axis view, preferably prior to the administration of contrast agents.

## Figures and Tables

**Table 1:** Imaging parameter of the cine sequences.

Parameter	bSSFP_ref_	CS_45_	CS_70_
Sequence	2D bSSFP cine	2D bSSFP cine	2D bSSFP cine
ECG mode	Retrospective	Retrospective	Retrospective
Field of view (mm^2^)	360 × 320	360 × 320	360 × 320
Matrix	192 × 171	192 × 171	192 × 171
Spatial resolution (mm^2^)	1.88 × 1.88	1.88 × 1.88	1.88 × 1.88
Slice thickness (mm)	8	8	8
Repetition time (ms)	3.12	2.86	2.86
Echo time (ms)	1.51	1.34	1.34
Flip angle(degrees)	45	45	70
Temporal resolution (ms)	31.2	42.9	42.9
Bandwidth (Hz/pixel)	1200	1200	1200
Cardiac phase (*n*)	25	25	25
Acceleration factor	2	11.4	11.4
Number of breath-hold ((short-axis, *n*)	9.1 ± 0.6	1	1
Iterative reconstruction (*n*)	-	80	80

CS_45/70_ = CS cine with 45/70° flip angle; bSSFP_ref_, referenced balanced steady state free precession; CS, compressed sensing.

**Table 2:** Demographic variables of the population (*n* = 104).

Characteristics	Patients (*n* = 104)	Range
Age (y)	46.5 ± 17.1	14–86
Sex (Female/Male)	29/75	-
Height (cm)	167.5 ± 8.9	146–188
Weight (kg)	68.8 ± 17.1	42–129
BMI (kg/m^2^)	24.3 ± 4.5	17.3–41.2
Main cardiovascular-related etiology		
HCM	24	-
Arrhythmia	23	-
ICM	17	-
DCM	13	-
Myocarditis	11	-
Hypertension	5	-
Amyloidosis	3	-
Takotsubo cardiomyopathy	2	-
Rheumatic heart disease	3	-
ARVC	1	-
Glycogen storage disease	1	-
Sarcoidosis	1	-

Data are reported as means ± SDs. BMI, body mass index; HCM, hypertrophic cardiomyopathy; ICM, ischemic cardiomyopathy; DCM, dilated cardiomyopathy; ARVC, arrhythmogenic right ventricular cardiomyopathy.

**Table 3:** Comparison of heart rate, scanning time, and image quality between every two cine sequences.

	bSSFP_ref_ ^a^ (Mean ± SD)	CS_45_ ^b^ (Mean ± SD)	eCS_45_ ^c^ (Mean ± SD)	eCS_70_ ^d^ (Mean ± SD)	*p* Value					
					ab	ac	ad	bc	bd	cd
Heart rate of short-axis cine (Bpm)	67.7 ± 11.8	67.8 ± 11.6	68.1 ± 12.0	67.8 ± 10.9	-	-	-	-	-	-
Scanning time of short-axis cine (s)	119.7 ± 23.3	18.2 ± 3.3	18.2 ± 3.3	18.2 ± 3.1	**	**	**	-	-	-
Scanning time of four chamber cine (s)	10.5 ± 2.4	1.8 ± 0.3	1.8 ± 0.3	1.8 ± 0.3	**	**	**	-	-	-
Global image quality (score)	2.8 ± 0.4	3.00 ± 0.00	2.9 ± 0.3	3.00 ± 0.0	**	*	**	*	-	*
Artifacts (score)	4.5 ± 0.7	4.9 ± 0.3	4.7 ± 0.5	4.9 ± 0.3	**	*	**	**	-	**
Image contrast (score)	2.5 ± 0.3	2.5 ± 0.3	1.7 ± 0.2	2.1 ± 0.3	-	**	**	**	**	**

Data are reported as means ± SDs. CS, compressed sensing; bSSFP, balanced steady state free precession. CS_45/70_ = CS-cine with 45/70° flip angle, e represents contrast enhanced. ^a^ = bSSFP_ref_, ^b^ = CS_45_, ^c^ = eCS_45_, ^d^ = eCS_70_, * = *p* < 0.05, ** = *p* < 0.001.

**Table 4:** Comparison of biventricular volume and function parameters between every two cine sequences.

	bSSFP_ref_ ^a^ Mean ± SD	CS_45_ ^b^ Mean ± SD	eCS_45_ ^c^ Mean ± SD	eCS_70_ ^d^ Mean ± SD	*p* Value					
					ab	ac	ad	bc	bd	cd
LVEDV (mL)	161.3 ± 65.5	159.4 ± 67.9	166.9 ± 69.2	165.0 ± 67.1	*	**	**	**	**	**
LVESV (mL)	88.5 ± 68.8	89.3 ± 70.8	93.4 ± 72.4	92.1 ± 70.2	-	**	**	**	**	*
LVSV (mL)	72.7 ± 26.0	70.0 ± 25.0	73.4 ± 24.9	72.9 ± 25.2	**	-	-	**	**	-
LVEF (%)	49.2 ± 16.9	48.3 ± 16.5	48.2 ± 16.1	48.2 ± 16.1	**	**	**	-	-	-
LVM (g)	120.8 ± 48.7	123.4 ± 49.8	119.2 ± 48.3	121.0 ± 49.1	**	*	-	**	**	**
RVEDV (mL)	137.4 ± 48.3	136.4 ± 49.1	148.7 ± 53.1	146.8 ± 48.4	-	**	**	**	**	-
RVESV (mL)	79.6 ± 38.0	79.5 ± 39.5	88.3 ± 44.1	86.6 ± 39.3	-	**	**	**	**	*
RVSV (mL)	57.8 ± 26.1	56.9 ± 25.8	60.4 ± 26.7	60.2 ± 26.0	*	**	**	**	**	-
RVEF (%)	42.9 ± 13.5	42.8 ± 13.5	41.8 ± 13.2	42.0 ± 13.0	-	**	**	**	**	-

Data are reported as means ± SDs. LVEDV, left ventricular end-diastolic volume; LVESV, left ventricular end-systolic volume; LVSV, left ventricular stroke volume; LVEF, left ventricular ejection fraction; LVM, left-ventricular mass in end-diastolic; RVEDV, right ventricular end-diastolic volume; RVESV, right ventricular end-systolic volume; RVSV, right ventricular stroke volume; RVEF, right ventricular ejection fraction; CS, compressed sensing; bSSFP, balanced steady state free precession. CS_45/70_ = CS-cine with 45/70° flip angle, e represents contrast enhanced. ^a^ = bSSFP_ref_, ^b^ = CS_45_, ^c^ = eCS_45_, ^d^ = eCS_70_, * = *p* < 0.05, ** = *p* < 0.001.

**Table 5:** Intra and inter-observer variability testing of the left ventricle using intraclass coefficient (ICC) for 20 randomly selected patients.

	Intra-Observer Variability				Inter-Observer Variability			
	bSSFP_ref_	CS_45_	eCS_45_	eCS_70_	bSSFP_ref_	CS_45_	eCS_45_	eCS_70_
LVEDV	0.983 **	0.980 **	0.986 **	0.980 **	0.974 **	0.977 **	0.978 **	0.969 **
LVESV	0.976 **	0.925 **	0.965 **	0.957 **	0.964 **	0.963 **	0.963 **	0.970 **
LVSV	0.959 **	0.955 **	0.932 **	0.926 **	0.964 **	0.905 **	0.906 **	0.903 **
LVEF	0.950 **	0.911 **	0.906 **	0.900 **	0.953 **	0.916 **	0.930 **	0.929 **
LVM	0.990 **	0.990 **	0.981 **	0.983 **	0.973 **	0.973 **	0.953 **	0.970 **

LVEDV, left ventricular end-diastolic volume; LVESV, left ventricular end-systolic volume; LVSV, left ventricular stroke volume; LVEF, left ventricular ejection fraction; LVM, left- ventricular mass in end-diastolic; CS, compressed sensing; bSSFP, balanced steady state free precession. CS_45/70_ = CS-cine with 45/70° flip angle, e represents contrast enhanced. ** = *p* < 0.001.

## Data Availability

The data presented in this study are available upon reasonable request and from the corresponding author.

## Supplementary Materials

The following supporting information can be downloaded at: https://www.mdpi.com/article/10.3390/jcm13030753/s1, Table S1: Intra-observer variability of left ventricle with 95% CI; Table S2: Inter-observer variability of left ventricle with 95% CI.
