# Supplementary material for: Optimizing Clinical Cardiac MRI Workflow through Single Breath-Hold Compressed Sensing Cine: An Evaluation of Feasibility and Efficiency
Source: J Clin Med. 2024 Jan 28;13(3):753. doi: 10.3390/jcm13030753 (PMC10856388; doi:10.3390/jcm13030753)
Supplement: Supplementary file 1 [file jcm-13-00753-s001.zip › jcm-2766017-supplementary.pdf]

Journal of
Clinical Medicine

MDPI

Table S1. Intra-observer variability of left ventricle with 95% CI.

	Intra-observer variability							
	**$bSSFP_{ref}$**	**95% CI**	**CS_{45}**	**95% CI**	**eCS_{45}**	**95% CI**	**eCS_{70}**	**95% CI**
LVEDV	0.983**	0.950–0.994	0.980**	0.915–0.993	0.986**	0.965–0.994	0.980**	0.937–0.993
LVESV	0.976**	0.941–0.991	0.925**	0.514–0.979	0.965**	0.901–0.987	0.957**	0.877–0.984
LVSV	0.959**	0.892–0.984	0.955**	0.891–0.982	0.932**	0.838–0.973	0.926**	0.823–0.970
LVEF	0.950**	0.880–0.980	0.911**	0.793–0.964	0.906**	0.775–0.962	0.900**	0.768–0.959
LVM	0.990**	0.975–0.996	0.990**	0.974–0.996	0.981**	0.950–0.993	0.983**	0.956–0.993

CI, credibility interval; LVEDV, left ventricular end-diastolic volume; LVESV, left ventricular end-systolic volume; LVSV, left ventricular stroke volume; LVEF, left ventricular ejection fraction; LVM, left- ventricular mass in end-diastolic; CS, compressed sensing; bSSFP, balanced free steady state precession. $CS_{45/70}$ = CS-cine with 45/70° flip angle, e represents contrast enhanced. ** = p <0.001.

Table S2. Inter-observer variability of left ventricle with 95% CI.

	Inter-observer variability							
	**$bSSFP_{ref}$**	**95% CI**	**CS_{45}**	**95% CI**	**eCS_{45}**	**95% CI**	**eCS_{70}**	**95% CI**
LVEDV	0.974**	0.937–0.990	0.977**	0.943–0.991	0.978**	0.946–0.991	0.969**	0.925–0.988
LVESV	0.964**	0.912–0.986	0.963**	0.902–0.986	0.963**	0.891–0.986	0.970**	0.925–0.988
LVSV	0.964**	0.904–0.986	0.905**	0.776–0.961	0.906**	0.775–0.962	0.903**	0.770–0.960
LVEF	0.953**	0.874–0.982	0.916**	0.802–0.966	0.930**	0.814–0.973	0.929**	0.829–0.971
LVM	0.973**	0.875–0.991	0.973**	0.878–0.991	0.953**	0.858–0.983	0.970**	0.923–0.988

CI, credibility interval; LVEDV, left ventricular end-diastolic volume; LVESV, left ventricular end-systolic volume; LVSV, left ventricular stroke volume; LVEF, left ventricular ejection fraction; LVM, left- ventricular mass in end-diastolic; CS, compressed sensing; bSSFP, balanced free steady state precession. $CS_{45/70}$ = CS-cine with 45/70° flip angle, e represents contrast enhanced. ** = p <0.001.
